# Exploring the probiotic landscape in understanding postbiotics from indigenous bacteria isolated from the stool samples of a tribal population at Mulluvadi village, Tamil Nadu, India

**DOI:** 10.3389/fphar.2026.1817531

**Published:** 2026-05-18

**Authors:** G. R. Shree Kumari, Mohanasrinivasan Vaithilingam

**Affiliations:** School of Bio Sciences and Technology, Vellore Institute of Technology, Vellore, Tamil Nadu, India

**Keywords:** gut health, Stool sample, postbiotics, probiotics, tribal gut microbiome

## Abstract

**Background:**

Urbanisation has been associated with a decline in the diversity of the gut microbiome that could potentially limit access to functionally robust probiotic strains. In contrast, traditional tribal populations represent underexplored reservoirs of diverse microbiota with unique metabolic capabilities. In this study, isolated and functionally characterised culturable probiotic bacteria from the gut microbiomes of individuals from the Mulluvadi tribal community (Tamil Nadu, India), with a focus on their inferred postbiotic-producing potential.

**Methods:**

A total of 112 microbial isolates were obtained from the stool samples of 25 healthy individuals, from which 13 representative bacterial strains were shortlisted by sequential screening based on phenotypic and functional criteria. These isolates were evaluated for tolerance to gastrointestinal stress conditions (pH, bile salts, NaCl, and temperature), cell surface hydrophobicity, auto-aggregation, safety attributes, and functional properties, including antibacterial activity, exopolysaccharide production, protease activity, biofilm formation, and short-chain fatty acid (SCFA) production.

**Results:**

The 13 isolates, mainly comprising *Lactiplantibacillus plantarum* and *Lacticaseibacillus rhamnosus*, exhibited >70% survival under simulated gastric and biliary conditions, high levels of hydrophobicity (60%–80%), strong inhibition of pathogens (12–25 mm), significant production of SCFAs, high levels of protease activity (15–20 mm clearance), and marked membrane stabilising effects of human red blood cells (65%–82%). All isolates were non-haemolytic, negative for DNase production, and displayed safety profiles consistent with those of probiotics. In particular, *L. plantarum* and *Heyndrickxia coagulans* were identified as the most functionally potent strains.

**Conclusion:**

Probiotic isolates from the gut microbiomes of a tribal population show remarkable postbiotic-producing capacity and potential functional relevance. These strains are promising candidates for further investigations toward the development of postbiotic-based functional formulations; however, their efficacies must be first established in animal and clinical trials and validated through additional *in vivo* and clinical studies for gut dysbiosis and other related disorders.

## 1 Background

Human wellbeing is heavily based on lifestyle factors, such as a balanced diet, regular physical activity, and a strong immune system, which endow the ability to continuously resist environmental microbes. The human gut microbiome benefits greatly from a diet rich in prebiotics and probiotics; in turn, a healthy gut microbiome produces short-chain fatty acids (SCFAs) that regulate inflammation and improve barrier function. The human body contains both beneficial and harmful microbes; in particular, the human gut contains an entire microbiome where beneficial taxa are selectively fuelled by prebiotics (Gibson and Roberfroid, 1995; Davani-Davari et al., 2019), while live microorganisms interacting with the resident immune cells and communities are often supported by probiotics (Lilly and Stillwell, 1965; El Hage et al., 2017). Certain preparations of inactivated micro-organisms and/or their components can confer health benefits to the host; this concept has been extended to deliver safe and standardised bioactive structures and metabolites as postbiotics while avoiding the risks associated with live-cell administration (Salminen et al., 2021; Shree Kumari and Mohanasrinivasan, 2025). Emerging evidence suggests that postbiotics may exert functions similar to probiotics in certain contexts, particularly immune modulation and epithelial protection, although their effects remain context-dependent; they also afford better stability, more predictable safety profiles, and easier formulations of foods and pharmaceuticals.

Tribal populations serve as valuable reservoirs for the discovery of robust postbiotic-producing probiotic strains because the gut microbiota of these individuals generally possess greater taxonomic diversity and functional richness than their urban counterparts. This is mainly attributable to their traditional diets, minimal consumption of ultra-processed foods, and different environmental microbial exposures (Vallianou et al., 2020; Salminen et al., 2021). The gut microbiomes of non-industrialised or tribal communities are notably enriched with fibre-degrading and SCFA-producing lineages, along with the occasional loss of configuration observed in metropolitan populations due to lifestyle changes (Dehingia et al., 2015); multiple studies from India and other regions with low urbanisation rates have reported these observations (Dehingia et al., 2015; Ramadass et al., 2017; Hazarika et al., 2022). Hence, our selection of a tribal community such as the Mulluvadi tribe in Tamil Nadu, India, as a source of stool isolates in the present study has strong scientific rationale because it increases the likelihood of discovering microbial strains that are not only capable of handling high-fibre diets but also resilient to environmental stresses, with metabolite profiles that are in agreement with host–microbe symbiosis/co-evolution (Pulipati et al., 2020). Within such a framework, extracting postbiotic-producing probiotic bacteria from the stool microbiota of the Mulluvadi tribal community can be considered a new approach as it combines ethno-microbiological sampling with a targeted functional screening pipeline focused on postbiotic output rather than simple description of the community composition (Tamang et al., 2022).

Recently, numerous studies have reported tribal gut microbial diversity based on sequencing surveys (Krishnan et al., 2026; Rishi et al., 2026), but only a handful of these works extend further to the cultures of such tribes to functionally characterise and position the candidate strains as postbiotic biotherapeutic resources (Torri, 2011; Chikindas et al., 2026), especially in the context of the tribal groups of south India. Therefore, the present work on “screening for postbiotic-producing probiotic bacteria from tribal stool samples” is a highly relevant yet timely contribution that is in agreement with emerging consensus definitions and regulatory interest in postbiotic preparations. Moreover, the identification of isolates that simultaneously exhibit anti-inflammatory protease activities with bacteriocin production, SCFA synthesis, exopolysaccharide (EPS) secretion, and biofilm-forming capacities would support their classification as potential next-generation probiotic candidates with postbiotic-producing capacities (Mafe et al., 2026; Pat et al., 2026). SCFAs such as acetates, propionates, and butyrates are vital for gut health as they provide energy to the colonocytes, enhance the epithelial barrier, and offer anti-inflammatory and antimicrobial properties. In addition, EPSs and biofilms support mucosal adhesion, immunomodulation, and colonisation resistance (Jadhav et al., 2026). Simultaneous production of bacteriocin and anti-inflammatory proteases also indicates the dual capacity to eliminate pathogens and reduce inflammation, which in turn opens up possibilities for functional foods, nutraceuticals, and targeted interventions in the treatment of chronic inflammatory or metabolic disorders. Therefore, the present study focused on screening stool samples from the Mulluvadi tribal community for microbial strains with potential multi-trait compartmentalised functional profiles and describing their postbiotic outputs as the main contributions; these novel approaches and data are expected to be useful in the rapidly expanding field of postbiotics and microbiome-based therapeutics. The present research not only helps understand the impacts of lifestyle and geography on the reservoirs of beneficial microbes but also offers a logical basis for producing safe and stable postbiotic formulations from culturally distinct and microbiologically rich populations.

## 2 Materials and methods

In this study, different incubation temperatures were employed for assay-specific optimisation, with 37 °C being used for the physiological conditions and 30 °C being used to support optimal growth for certain *in vitro* assays.

### 2.1 Ethical procedures

The procedures for collecting stool samples from the Mulluvadi tribal population received ethical approval from the Institutional Ethics Committee on Human Studies at Vellore Institute of Technology (VIT/IECH/2025/16/IECH, dated 15 February 2025). Blood samples were obtained from the human subjects for the haemolytic assays and identification of pathogenic indicator strains (*Pseudomonas aeruginosa* MTCC 2582, *Escherichia coli* MTCC 443, *Listeria monocytogenes* MTCC 657, and *Staphylococcus aureus* MTCC 3160); these samples were also used for the probiotic screening and were granted ethical clearance (VIT/IECH/2025/16 IECH) and Institutional Biosafety Committee approval (VIT/IBSC9/03). Informed consent was obtained from all participants in the regional language (Tamil) after explaining the purpose, procedures, and voluntary nature of the study. The study followed all ethical guidelines established by The Indian Council of Medical Research 2017 (ICMR) and principles of the Declaration of Helsinki. The consent form also explained the participant right to remain anonymous and withdraw freely from the study at any time.

### 2.2 Sample size determination

Stool samples (n = 25) were collected from healthy individuals of the Mulluvadi tribal community, who constitute approximately 5%–10% of the estimated healthy population based on previous community-based surveys. The sample size was determined using prevalence-based power calculations, which are standard for probiotic and microbiome isolation studies (Ramadass et al., 2017; Hazarika et al., 2022). The expected prevalence of carrying *Lactobacillus spp*. was set at 30%, which is consistent with reports from populations consuming traditional high-fibre diets as opposed to the lower prevalence commonly found in urban populations (5%–15%). The minimum number of samples required was calculated using the standard formula for prevalence studies:

$$n=\frac{z^{2}p\times \left(1-p\right)}{d^{2}}.$$

Here, we considered z = 1.96, *p* = 0.30, and d = 0.10 to obtain the final size of the study group as 25 participants; this number is adequate for exploratory isolation and screening studies based on comparable microbiome research, as per the ICMR guidelines for microbiome research.

### 2.3 Sample collection

Stool samples were collected from individuals residing at Mulluvadi tribal village (12.8067° N, 79.4302° E). A total of 25 stool samples were gathered from healthy individuals of the tribal community aged between 5 and 70 years. These participants are considered to capture the representative microbial diversity within the tribal population given the logistical constraints of stratified sampling. The stool samples of the participants were collected using sterile stool container specimen cups with spoon lid (50 mL). Following collection, the samples were transported to the laboratory in an ice box within 1.5 h. The guidelines established by the ICMR were followed for sample collection, while the guidelines established by Kenbiolinks Pvt Ltd. were followed for biowaste disposal throughout the study.

### 2.4 Sample processing

Upon arrival at the iProzymes TT-121 Laboratory, Vellore Institute of Technology, Vellore, Tamil Nadu, India, the samples were segregated and assigned the codes VITFS01–VITFS25 to maintain confidentiality and consistency throughout the study. Each stool sample was then weighed for approximately 1 g under highly sterile conditions and carefully inoculated into an enrichment medium (DebMandal et al., 2012; Srijan et al., 2015) comprising the de Man, Rogosa, and Sharpe (MRS) broth for 24 h at 37 °C in a shaker incubator; later, each sample was diluted serially and plated onto MRS agar medium using the pour plate technique and incubated anaerobically. All experiments were performed in triplicate unless specified otherwise, and appropriate negative and positive controls were included for each assay.

### 2.5 Isolation and preliminary characterisation of potential probiotic strains from stool samples

Based on the colony morphology, the isolates were streaked onto MRS agar, purified through repeated sub-culturing, and assessed for Gram’s reaction (Jacobson, 1983) and catalase and oxidase activities (Khushboo et al., 2023). Isolates exhibiting Gram-positive rods or spherical cellular morphologies were considered for the phenol red and calcium carbonate plate assays to assess organic acid production.

#### 2.5.1 Phenol red assay to assess production of organic acids

MRS broth supplemented with 2% glycerol and 0.002% (w/v) phenol red (MRSG + P) indicator dye was used to identify the micro-organisms synthesising organic acids. Approximately 100 µL of each isolate grown for 16 h in MRS broth and producing approximately 4 × 10^8^ CFU/mL using the McFarland standard were inoculated into the MRSG + P broth and incubated anaerobically for 12 h at 37 °C, with the uninoculated MRSG + P broth being used as control. After incubation, the optical density (OD) values were determined at 560 nm. Phenol red was used as the pH indicator for detecting bacterial fermentation (Qureshi et al., 2022; Elango et al., 2024).

#### 2.5.2 Calcium carbonate plate assay

Bacterial isolates exhibiting Gram-positive (rods/cocci) results were freshly streaked onto MRS agar plates supplemented with 0.1% calcium carbonate and incubated anaerobically at 37 °C for 24–48 h to observe the zone of clearance (Siddique and Vaithilingam, 2023).

### 2.6 Probiotic characterisation

#### 2.6.1 Acid, NaCl, bile salt, and temperature tolerances

The viability of the selected isolates in the presence of gastrointestinal stressors was determined by subjecting them to a wide range of pH, osmotic, biliary, and thermal conditions. Cultures at the seed stage (200 μL) were used to inoculate 10 mL of MRS broth with the pH adjusted to 2, 4, 6, 8, and 10; with NaCl concentrations of 2%, 4%, 6%, 8%, and 10%; and with the addition of bile salts at 0.2%, 0.4%, 0.6%, 0.8%, and 1%, where the untreated sample was considered the control in each case. The initial bacterial titre (CFU/mL) was determined at the zeroth hour using the spreading plate method; then, a 200 μL sample from each tube was transferred into a 96-well plate and the OD was measured at a wavelength of 550 nm (OD550) using a microplate spectrophotometer to assess growth within the first hour. After incubating the tubes for 3 h at 37 °C, the CFU and OD550 values were measured during the third hour to quantify survival and growth. Temperature resistance is an important characteristic of a probiotic considering its product manufacturing and intestinal transit; hence, tests were performed similar to the above conditions by growing the isolates in MRS broth at 10 °C, 21 °C, 37 °C, and 45 °C for 48 h, followed by monitoring the growth via turbidity to determine the viable cells (Deshpande and Bhate, 2017; Kook et al., 2019; Khushboo et al., 2023).

#### 2.6.2 Cell surface hydrophobicity and cell auto-aggregation

Promising isolates were cultured in MRS broth at 30 °C for one day and collected using a centrifuge (5,000 rpm for 10 min at 4 °C); thereafter, the cells were washed twice in phosphate-buffered saline (PBS), resuspended in PBS up to a volume of 6 mL, and initial OD600 was measured. The cell surface hydrophobicity, which is the main factor affecting epithelial adherence, was evaluated as follows: a 3 mL aliquot of the cell suspension was mixed with 1 mL of n-hexane, vortexed intensively for 2 min, and left for layer separation before recording the final OD at 600 nm. The percentage hydrophobicity is defined as $\left(1-\frac{OD\;initial\;}{OD\;final\;}\;\right)\times 100$ and shows the affinity of solvent partitioning (Tamilselvan et al., 2026). Furthermore, cell auto-aggregation (Suwannaphan, 2021; Tamilselvan et al., 2026) was similarly determined as a feature suggesting the potential adhesion-related traits under *in vitro* conditions: cells from the MRS culture were first incubated overnight and centrifuged (5,000 rpm for 10 min at 4 °C); then, the obtained pellets were washed twice with PBS, resuspended in 6 mL of PBS, and measured for OD600 at t = 0. The suspensions were next incubated without shaking for 2 h, measured for the final OD600, and used to derive the percentage of aggregation as $\left(1-\frac{OD2h\;}{OD0h}\;\right)\times 100$ .

#### 2.6.3 Probiotic resistance to gastric acidity

The gastric acid tolerances of the bacterial strains were evaluated as a model of their passage through the stomach (Casarotti et al., 2015). In brief, the bacterial cultures were incubated overnight in MRS medium and centrifuged; the cell pellets obtained thus were resuspended in PBS (pH 7), and the cell suspensions were inoculated (1% v/v) into MRS broth at pH 2.5 by adjusting with 1 M HCl and 0. 3% pepsin, followed by incubation at 37 °C for 34 h. The numbers of surviving bacteria (CFU/mL) were determined before and after exposure through serial dilution and plating on MRS agar, with the survival rate being calculated as follows:

$$\frac{\log\left(\text{CFU}\;\text{post}-\text{exposure}\right)}{\log\left(\text{CFU}\;\text{initial}\right)}\times 100.$$

Strains that were able to maintain more than 50% viability showed sufficient resilience to the gastric environment that was deemed compatible with oral delivery.

### 2.7 Safety assessments

#### 2.7.1 Haemolytic activity

One of the crucial parameters for assessing the safety of a potential isolate is its haemolytic potential. A greenish discolouration spreading from the colonies indicates α-haemolysis, while clear lysis zones reveal β-haemolysis; the absence of a reaction is considered as γ-haemolysis and is a safe phenotype for probiotics. To assess the haemolytic activities of the isolates, we used blood agar base (HiMedia, India) substituted with 5% human blood (Archi and Juhana, 2023).

#### 2.7.2 Extracellular DNase activity

Selected bacterial isolates were streaked on DNase test agar with methyl green (Sisco Research Laboratories Pvt Ltd., India) and incubated aerobically at 37 °C for 48 h. Following incubation, the plates were examined for transparent zones around the colonies that indicate digestion of DNase. If no zones of this kind were found, then it meant that the bacteria did not have harmful properties; this outcome is in line with the safety standards of probiotic selection (Gopal and Dharumadurai, 2022; Archi and Juhana, 2023).

#### 2.7.3 Susceptibility of cultures to antibiotics

The susceptibility profiles of the isolates against antibiotics were determined by the disc diffusion method on MRS agar (Temmerman et al., 2003; Zhou et al., 2005). The log-phase cultures were uniformly lawned across the entire surfaces of the plates; then, commercial discs impregnated with streptomycin (S), vancomycin (VA), clindamycin (CD), chloramphenicol (C), erythromycin (E), azithromycin (AZM), gentamicin (GEN), ciprofloxacin (CIP), ampicillin (AMP), and penicillin-G (P) were placed on the plates. The discs were allowed to pre-diffuse at 4 °C for 30 min before incubation at 30 °C for 48 h; the diameters of the zones were measured and interpreted as susceptible, intermediate, or resistant.

### 2.8 Screening for postbiotics

#### 2.8.1 SCFA production

##### 2.8.1.1 Medium for SCFA production

The following were the components of the medium used to produce SCFAs (Zhang et al., 2023): 27.57 g/L of MRS broth, 15 g/L of prebiotic oats powder (Quaker Oats), 1.5 g/L of bile salts, 10 g/L of maltose, 0.1 g/L of CaCO_3_, and 2% glycerol; the medium was prepared and autoclaved for 15 min at 121 °C and 15 psi. Once the fermentation broth cooled to room temperature, a 5% solution of the inoculum was added to the fermentation medium under sterile conditions and incubated anaerobically at 37 °C for 72–96 h.

##### 2.8.1.2 Screening for SCFAs using ultra-performance liquid chromatography with photodiode array detection method

Following incubation, the cultures were transferred to a 50 mL centrifuge tube aseptically and spun at 8,000 rpm and 4 °C for 15 min. As the supernatant is of interest in this study, we focused on screening only three important SCFAs, namely, acetic acid, butyric acid, and propionic acid. Thus, we screened the supernatant for the presence of acetic, propionic, and butyric acids as an indicator of the SCFA-producing potential; the analysis was considered qualitative to semi-quantitative via the ultra-performance liquid chromatography (UPLC)–ultraviolet (UV) method against the respective standards (Calvigioni et al., 2023).

###### 2.8.1.2.1 Standard and sample preparation to detect SCFAs using UPLC with UV analysis

We procured an SCFA kit (SBR00030, Merck Sigma-Aldrich) containing sodium acetate (1 M), sodium propionate (0.5 M), sodium butyrate (0.5 M), and mixed SCFAs (1 mL) as per the required standards. To prepare the standard and samples, we used the following derivatisation technique to convert the carboxylate salts to their corresponding free acids by acid hydrolysis using acetone and 2,3,4,5,6-pentafluorobenzyl bromide (PFB-Br; 90257, Merck Sigma-Aldrich) (Merck KGaA, Darmstadt, Germany). The derivatisation reaction for each component was prepared as provided in the safety data sheet. Each mixture was prepared homogeneously in a glass screw-cap vial according to the composition shown in Table 1; the solution mixture was then heated to 65 °C for 24 h with the cap closed to complete the reaction.

**TABLE 1:** Derivatisation of the reaction mixtures.

Component	Volume utilised	Acetone	PFB-Br
Acetate (sodium acetate 1 M)	100 µL	900 µL	15 µL
Propionate (sodium propionate 0.5 M)	200 µL	800 µL	15 µL
Butyrate (sodium butyrate 0.5 M)	200 µL	800 µL	15 µL
Short-chain fatty acid mix	100 µL	900 µL	3 µL
Cell-free supernatant collected from each isolate	1 mL	4 mL	15 µL

###### 2.8.1.2.2 Chromatographic conditions and analysis of SCFAs

The chromatographic system used for the analysis was a Waters ACQUITY UPLC H-Class device with a photodiode array (PDA) detector. An Ascentis® Express C18 UPLC column of length 100 mm and internal diameter 2.1 mm with a particle size of 1.7 μm; the column oven was set to maintain a constant temperature of 25 °C. A flow rate of 0.2 mL/min was used for the mobile phase (Bihan et al., 2022; McKay et al., 2023). The analytes were detected at a wavelength of 210 nm, and the injection volume for each sample was 1 µL. The gradient elution parameters used are detailed in Table 2. This analysis allows preliminary detection of SCFAs; however, full analytical validation including calibration curves, limits of detection, and recovery efficiency was not performed and represents a limitation of our study.

**TABLE 2:** Recommended solvent gradients for the UPLC–PDA analysis.

Time (min)	Acetonitrile (%)	Water + 0.1% trifluoroacetic acid
0	20	80
15	80	20
18	20	80
20	20	80

#### 2.8.2 Enzyme production: protease activity and membrane stabilisation potential (*in vitro* proxy for anti-inflammatory activity)

##### 2.8.2.1 Protease activity on skim milk agar

An skim milk agar (SMA) base with 0.5% NaCl, 0.5% peptone, and 1.5% agar in distilled water (pH 6.8–7.0) was autoclaved (121 °C, 15 min), and sterile 10% skimmed milk prepared by sequential heating at 85 °C (30 min) and 100 °C (10 min) was added aseptically to the mixture after cooling to 50 °C–55 °C before being poured into plates and allowed to solidify. The protease activity was detected by diffusing the cell-free supernatant (100 µL) in 6 mm wells punched using a sterile cork borer; the plates were then pre-chilled at 4 °C for 10 min prior to anaerobic incubation, and the halo diameters were measured to quantify the extracellular casein hydrolysis (Devi et al., 2018).

##### 2.8.2.2 Membrane stability assay using human red blood cells

Blood samples collected from healthy donors who were not under the effects of any non-steroidal anti-inflammatory drugs were mixed 1:1 with Alsever’s solution (Sigma-Aldrich) and centrifuged (3,000 rpm for 5 min); the obtained human red blood cells (HRBCs) were washed thrice with 0.9% saline to prepare a suspension of 10% (v/v) HRBCs in PBS (pH 7.4). Next, the test supernatant (1 mL), PBS (1 mL), hyposaline (0.5 mL, 0.35% NaCl), and HRBCs (0.5 mL) were incubated at 37 °C for 30 min before being centrifuged at 3,000 rpm for 20 min. Haemoglobin release was quantified by measuring the absorbance (Abs) of the supernatant at a wavelength of 560 nm (Devi et al., 2018), where diclofenac was used as the positive control and a hyposaline solution was used as the lysis control. The protection and haemolysis percentages were then calculated using the following formulae:

$$\text{Protection }\left(\%\right)=100\times \left(1-\frac{{\text{Abs}}_{\text{sample}}-{\text{Abs}}_{\text{control}}}{{\text{Abs}}_{\text{lysis}}-{\text{Abs}}_{\text{control}}}\right),$$

% Haemolysis=100−%Protection.

#### 2.8.3 EPS production

EPS production was assessed both qualitatively and quantitatively. Accordingly, a simple preliminary screening was conducted for EPS production by streaking the bacterial isolates on MRS agar plates enriched with 2% Congo red as the indicator. Here, the colonies appearing black on the MRS-Congo-red plates were considered as the best EPS producers, while colonies exhibiting brownish to red colour were categorised as moderate EPS producers and those appearing whitish or without black/brown/red colouration were confirmed as non-EPS producers. These strains were inoculated into sucrose-supplemented MRS broth and incubated at 37 °C for 48 h. Thereafter, the cells were removed by centrifugation, and the crude EPSs were extracted from the cell-free supernatant by adding two volumes of cold ethanol and incubating overnight at 4 °C. The yield was quantified by the phenol sulphuric acid assay (λ = 490 nm, glucose standards), and a ropy phenotype on agar confirmed slime-forming EPSs associated with immunomodulation and gut persistence (Devi et al., 2018; Taj et al., 2022).

#### 2.8.4 Biofilm production

The biofilm-forming capacity was evaluated by the crystal violet (CV) microtiter plate assay. Here, 96-well plates containing MRS broth were inoculated with the isolates (OD600 of ∼0.1) and allowed to adhere via static incubation at 37 °C for 24 h. The cells that did not adhere were washed away; the biofilms were then fixed with methanol, stained with 0.1% CV for 15 min, washed, and solubilised in 33% acetic acid before measuring the absorbance at 595 nm and the biomass compared to the control. The OD values were used to group the strains into non-producers (OD < 0.1) or weak (0.1–0.5), moderate (0.5–1.0), and strong (>1.0) biofilm producers (Sadeghi et al., 2022; Taj et al., 2022).

#### 2.8.5 Antibacterial activity

The inhibitory potential of each isolate against bacterial pathogens was determined by the agar well diffusion method. First, the isolated cultures were grown in MRS broth at 30 °C for 48 h, following which the cells were centrifuged at 8,000 rpm for 20 min, and the cell-free supernatants were collected and tested for their abilities to inhibit the growths of *P. aeruginosa* (MTCC 2582), *E. coli* (MTCC 443), *L. monocytogenes* (MTCC 657), and *S. aureus* (MTCC 3160). In brief, each indicator strain was grown overnight and uniformly swabbed on Mueller–Hinton agar plates; then, 6-mm-diameter wells were bored in each plate, filled with 100 µL of the supernatant, and incubated at 37 °C for 18–24 h. The inhibition zones were measured in the diametric direction, where ciprofloxacin served as the positive control and un-inoculated MRS broth was used as the negative control to ensure the specificity of the test (Samot and Badet, 2013; Cizeikiene and Jagelaviciute, 2021).

### 2.9 Gene sequencing using 16S rRNA

Genomic DNA samples were isolated from the selected probiotic strains by a conventional phenol-chloroform method with some modifications to process Gram-positive bacteria. The quality and concentration of the extracted DNA sample were assessed prior to downstream applications (Chomczynski and Sacchi, 2006). Purified genomic DNA was used as the template for amplifying the bacterial 16S rRNA gene (∼1.5 kb) using universal primers: 27F (5′-AGA​GTT​TGA​TCC​TGG​CTC​AG-3′) and 1492R (5′-TAC​CTT​GTT​ACG​ACT​T-3′). Polymerase chain reaction (PCR) amplification was performed in a thermal cycler under the following conditions: initial denaturation at 96 °C for 20 s, followed by 30 cycles of denaturation at 96 °C for 20 s each, annealing at 50 °C for 30 s, extension at 60 °C for 4 min, and a final extension at 60 °C for 10 min. The amplified products were resolved on 1% agarose gel prepared in 1× TAE buffer, and band visualisation was carried out using a gel documentation system. The PCR amplicons were next purified and subjected to Sanger sequencing using internal primers targeting the conserved regions of the 16S rRNA gene on an automated ABI DNA sequence. The raw chromatograms were base-called, trimmed, and quality-checked using ChromasPro software (Altschul et al., 1990). High-quality sequences were aligned using Clustal X (version 2.0), and the preliminary taxonomic identification was performed by querying the sequences against the GenBank database using BLASTn algorithm. Species-level identifications were assigned based on a sequence similarity threshold of ≥97%. For the phylogenetic analysis, the aligned sequences were imported into MEGA (version 12); then, the evolutionary relationships were inferred using the neighbour-joining method, where the genetic distances were calculated according to the Kimura two-parameter model (Tamura et al., 2007). The robustness of the phylogenetic tree was evaluated by bootstrap analysis with 500 replicates. Here, ambiguous nucleotide positions were excluded using the pairwise deletion option, and the branch lengths were scaled to represent the number of substitutions per site (Tamura et al., 2021; Veeruraj et al., 2026).

### 2.10 Statistical analysis

All experiments were performed in triplicate, and the quantitative data derived from the tolerance, activity, and functional assays were expressed in terms of the mean and standard deviation. Statistical analyses were performed using GraphPad Prism (version 10.6.1.892). The data were input as grouped datasets, and the data distributions were checked for normality using the Shapiro–Wilk test before performing the parametric analyses. To examine the differences, we applied two-way ANOVA where appropriate to evaluate the effects of the experimental conditions and isolates, followed by Dunnett’s multiple comparisons test against the control groups. Comparisons were also made within each row to evaluate the differences among the columns (simple effects within rows), in line with grouped experimental designs. Dunnett’s multiple comparisons test was employed as a *post hoc* procedure to compare the treatment groups with the corresponding controls. A *p*-value of <0.05 was deemed statistically significant. For all graphical data, the statistical significances are indicated as *p* < 0.05 (*), *p < 0.01* (**), p < 0.001 (***), and *p* < 0.0001 (****).

## 3 Results

### 3.1 Isolation and preliminary characterisation of potential probiotic strains from stool samples

**FIGURE 1:**
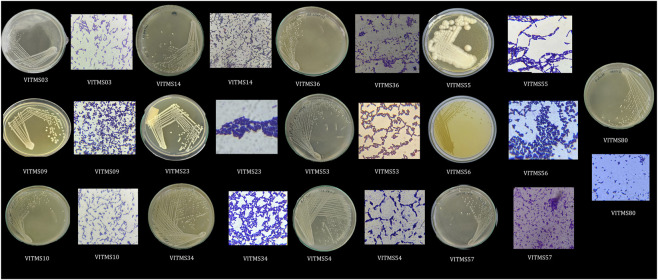
Morphological and Gram reaction characterisation of the probiotic isolates. Representative images showing pure culture colony morphologies and the corresponding Gram-stained microscopic images of the thirteen probiotic isolates.

**FIGURE 2:**
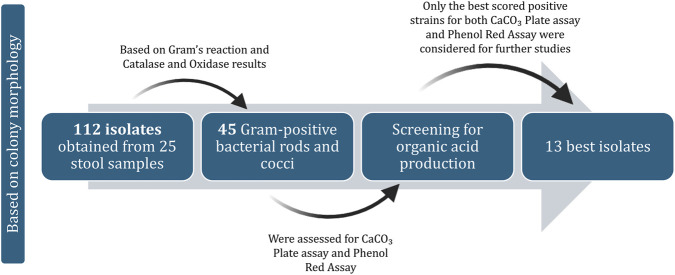
Isolate selection pipeline showing the inclusion and exclusion criteria.

Based on distinct colony morphologies, we obtained a total of 112 microbial isolates from the stool samples of 25 participants; of these, 75 were bacterial and 42 were yeast isolates. In the present study, only bacterial isolates were considered. Following Gram’s reaction and the catalase and oxidase tests, 45 Gram-positive rods and cocci exhibiting catalase- and oxidase-negative characteristics and two strains with catalase-positive and oxidase-negative characteristics were considered for further studies, while the remaining isolates were eliminated (Supplementary Table S1). The 45 bacterial isolates retained herein were tested for organic acid production using phenol red and calcium carbonate plate assays (Supplementary Figure S1). As a result of acidification by the organisms producing organic acids in the phenol red assay, the colour of the medium changed from red to orange/yellow (Supplementary Figure S2); samples showing clear halo zones in the calcium carbonate plate assay were scored for further study: VITMS03, VITMS09, VITMS10, VITMS14, VITMS23, VITMS34, VITMS36, VITMS53, VITMS54, VITMS55, VITMS56, VITMS57, and VITMS80. These 13 bacterial strains (Figure 1) were considered for the remainder of this study. Figure 2 exhibits the pipeline for selecting these 13 isolates based on sequential screening criteria, including Gram-positive morphology, absence of catalase and oxidase activities, and best positive organic acid production assays; only the best-scored positive strains for both the CaCO_3_ plate and phenol red assays were considered for further studies.

### 3.2 Probiotic characterisation

#### 3.2.1 Tolerance to gastrointestinal and environmental stresses

**FIGURE 3:**
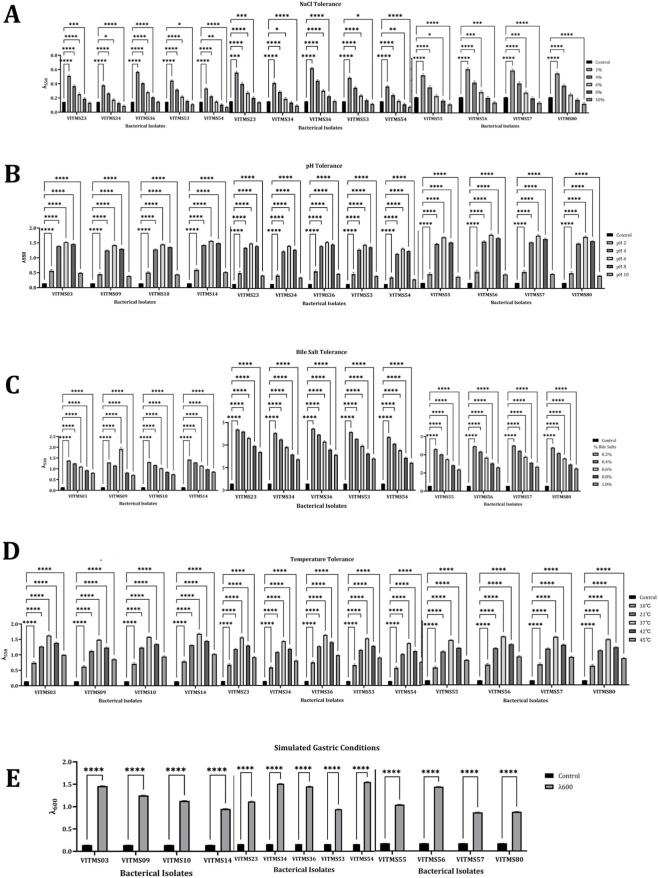
Gastrointestinal stress tolerance profiles of the probiotic isolates. Survival of the selected strains under varying **(A)** NaCl levels, **(B)** pH levels, **(C)** bile salt concentrations, and **(D)** temperature conditions is shown, along with **(E)** viability under simulated gastric conditions.

All 13 probiotic bacterial isolates were tested for tolerance to acids, NaCl, bile salts, and temperature as markers of their ability to survive the gastrointestinal tract and functional robustness, as presented in Figure 3. The acid tolerance test showed that VITMS03, VITMS10, VITMS36, VITMS53, and VITMS55 were able to retain high levels of viability even after 3 h of incubation compared to the other isolates, indicating their relatively higher tolerances to simulated *in vitro* gastric conditions (Figure 3B). The NaCl tolerance test showed that the responses of the isolates to osmotic stress differed depending on the isolate (Figure 3A). Here, a few strains (VITMS55, VITMS23, and VITMS10) continued to grow even in the presence of very high salt concentrations; thus, these strains have effective osmoregulatory systems that suggest their potential suitability for applications involving higher osmotic conditions. The bile salt tolerance test showed that isolates VITMS03, VITMS10, VITMS23, and VITMS55 were able to survive exposure to bile salt concentrations of physiological relevance; these strains showed sustained growth, which implies that they possess better capacity to cope with bile-induced membrane damage in the intestinal environment (Figure 3C). All isolates showed growth at 37 °C, which means that they are physiologically compatible with the temperature of the human body and are potential probiotics; however, VITMS53 and VITMS55 were also found to perform optimally at 45 °C (Figure 3D).

#### 3.2.2 Cell surface hydrophobicity and cell auto-aggregation

There were significant differences between the 13 bacterial isolates in terms of cell surface hydrophobicity and auto-aggregation, both of which are major factors that determine probiotic adhesion, colonisation, and retention in the gastrointestinal tract (Table 3). The measured cell surface hydrophobicity values were very high in some of the strains; accordingly, these strains displayed high hydrophobic solvent affinity (towards n-hexane) and also had a higher possibility of adhering to the epithelial and mucosal cells of the intestine. The three strains VITMS36, VITMS03, and VITMS10 showed the highest cell surface hydrophobicity values, whereas VITMS53 and VITMS55 showed moderate-to-high values, consistent with their probiotic potential. However, the hydrophobicity indices of VITMS23, VITMS80, and VITMS56 were very low to moderate, indicating poor ability to interact with the intestinal epithelium.

**TABLE 3:** Cell surface hydrophobicity and auto-aggregation properties of the probiotic isolates obtained from the stool samples of the Mulluvadi tribal population.

Strain	% Hydrophobicity	% Auto-aggregation	Standard deviation
VITMS09	78.5	66.1	2.1
VITMS23	82.3	61.5	1.8
VITMS34	71.2	60.8	2.4
VITMS55	65.8	60.2	2.7
VITMS56	76.9	59	2
VITMS80	69.4	51.4	2.5
VITMS03	85.7	50.2	1.5
VITMS10	79.1	49.2	2.2
VITMS14	88.2	47.6	1.3
VITMS36	83.6	45.3	1.7
VITMS53	74.3	45	2.3
VITMS54	62.1	40	2.9
VITMS57	77.8	38.9	2.1

#### 3.2.3 Probiotic resistance to gastric acidity

The survival capabilities of the 13 probiotic isolates when exposed to simulated gastric conditions (SGCs) were assessed by tracking the changes in OD upon 3 h of exposure to acidic conditions, as shown in Figure 3E. All isolates showed decreases in growth compared to their initial values, thus highlighting their sensitivity to gastric stress. Nevertheless, the degree of reduction differed significantly among the strains. In particular, VITMS10 and VITMS03 were identified to have the highest survival rates under SGCs out of the isolates tested, as indicated by their relatively higher OD values after 3 h of exposure. Further, VITMS36 and VITMS55 revealed certain degrees of resistance, suggesting that these isolates have better ability to endure gastric acidity; thus, these isolates have the greatest likelihood of surviving the transit through the stomach and reaching the intestine in a viable manner. Conversely, VITMS57 and VITMS80 showed the highest decreases in OD, confirming their poor survival under SGCs. Table 4 presents the survival percentages of the 13 postbiotic isolates after 3 h of exposure to SGCs. Here, lower tolerances imply decreased resistances to acidic stress and reduced probabilities of surviving the gastrointestinal transit. Upon general consideration, the isolate-specific differences in the ability to withstand SGCs emphasise the considerable variation in the degree of gastric stress resistance among the probiotic candidates.

**TABLE 4:** Survival of probiotic isolates under simulated gastric conditions.

Isolate	Mean optical density (post-exposure)	Survival (%) based on CFU level
VITMS03	1.462	97.50
VITMS09	1.130	75.30
VITMS10	1.253	83.50
VITMS14	0.951	63.40
VITMS23	0.862	57.50
VITMS34	1.349	89.90
VITMS36	1.295	86.30
VITMS53	0.840	56.00
VITMS54	1.385	92.30
VITMS55	0.995	66.30
VITMS56	1.194	79.60
VITMS57	0.718	47.90
VITMS80	0.729	48.60

### 3.3 Safety assessments

Assessment of the safety parameters is essential for commercialisation of probiotic bacteria for their postbiotic effects toward their hosts. To address these safety considerations, the 13 probiotic bacteria were assessed for production of enzymes such as haemolysin and DNase and susceptibility to 10 different antibiotics via the disc diffusion method (Supplementary Figure S3; Supplementary Table S3). None of the isolates produced haemolysin (no zones) on 5% human blood agar, thus confirming the lack of haemolytic virulence. There were no clear ring halos on the DNase test agar with methyl green (Sisco Research Labs); hence, the isolates were verified as non-DNase producers. As shown in Figure 6B, the isolates reveal susceptibility (zones in mm) to streptomycin (S), vancomycin (VA), clindamycin (CD), chloramphenicol (C), erythromycin (E), azithromycin (AZM), gentamicin (GEN), ciprofloxacin (CIP), ampicillin (AMP), and penicillin-G (P); furthermore, intrinsic resistance to vancomycin was observed in the VITMS56 strain. No multi-drug-resistant profiles were detected in any of the strains. These results reveal that the isolates are safe and would not cause any harm to the host on possible administration.

### 3.4 Screening for postbiotics

#### 3.4.1 Screening for SCFAs using UPLC–PDA detection method

**FIGURE 4:**
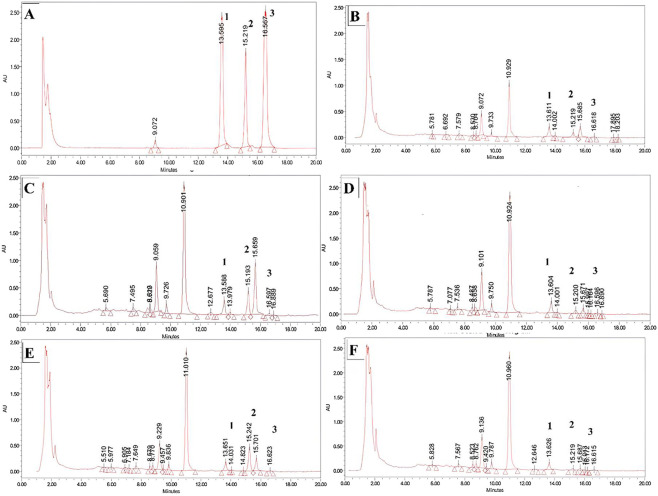
UPLC–PDA results for the production of **(A)** standard short-chain fatty acids (SCFAs) such as acetic acid (1), propionic acid (2), and butyric acid (3). SCFA production results for the isolates **(B)** VITMS03, **(C)** VITMS10, **(D)** VITMS57, **(E)** VITMS55, and **(F)** VITMS36.

The 13 bacterial isolates were tested for SCFA production. In brief, following anaerobic incubation at 37 °C for 96 h, the fermented broth was centrifuged at 8,500 rpm for 20 min, and the fresh supernatant obtained from each strain was derivatised as noted in Table 1 for UPLC–PDA analysis; these results are shown in Figure 4. The derivatised cell-free supernatant samples were individually analysed by UPLC–PDA to check for the production of SCFAs, mainly, acetates, propionates, and butyrates. Here, 5 out of the 13 isolates (VITMS03, VITMS10, VITMS36, VITMS55, and VITMS57) showed production of SCFAs when compared against the standard SCFAs.

#### 3.4.2 Enzyme production: anti-inflammatory protease activity and membrane stability potential

##### 3.4.2.1 Protease activity on SMA

**FIGURE 5:**
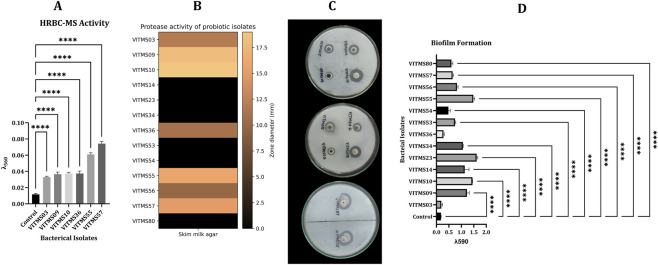
Composite representation of the postbiotic functional assays showing **(A)** membrane stabilisation of human red blood cells (HRBC-MS) expressed as percentage of membrane protection; **(B)** proteolytic activities visualised as a heatmap of the diameter of the zone of clearance (mm); **(C)** representative skim milk agar plate assays confirming extracellular protease production based on clear halo formation; **(D)** biofilm production quantified by the crystal violet microtiter plate assay, indicating strain-specific biofilm-forming capacity. The data represent mean values obtained from triplicate experiments.

Protease production by the probiotic isolates was first assessed with SMA using the well diffusion method for cell-free supernatants. Accordingly, VITMS09, VITMS10, VITMS36, VITMS55, and VITMS57 demonstrated strong proteolytic activities based on their visible clear zones, which indicate that these strains secreted extracellular proteases to create zones varying in diameter between 8 mm and 15 mm. These findings confirm that the isolates can efficiently hydrolyse milk proteins (Figures 5B,C).

##### 3.4.2.2 Membrane stability assay using HRBCs

The anti-inflammatory properties of the extracted proteases were assessed, and the percentage of membrane protection against hypotonicity-induced lysis was found to range from 65% to 90% for the supernatants. The effectiveness of diclofenac as an anti-inflammatory standard drug (97%) closely matches the degree of protection observed herein, implying that the enzymes produced by the isolates have significant anti-inflammatory properties (Figure 5A).

#### 3.4.3 EPS synthesis evaluation

EPSs synthesised by the probiotic strains were estimated by the MRS + Congo red plate and phenol sulphuric acid methods. Preliminary screening for EPS production was assessed by the MRS + Congo red plate method (Supplementary Figure S1B). The isolates VITMS09, VITMS14, VITMS23, VITMS53, VITMS54, and VITMS57 exhibited brownish coloured colonies indicating that they were moderate producers of EPSs; the remaining 7 out the 13 bacterial isolates were found to be strong producers of EPSs based on the blackish colonies formed on the assay plates. Furthermore, all 13 isolates were assessed for EPS synthesis by the phenol sulphuric acid method (Table 5). Specifically, VITMS10, VITMS36, and VITMS55 showed the highest EPS production levels of 20–25 μg/L, which reflect their robust polysaccharide biosynthetic capabilities. High levels of EPS secretion by these strains were also evidenced by their ropy phenotypes on sucrose-enriched MRS agar, which is an indicator of extracellular polysaccharide secretion. The remaining isolates generally produced lower quantities of EPSs and lacked the ropy phenotype, implying weak EPS synthesis ability. Strains producing high levels of EPSs are thus interesting owing to the well-documented functions of EPSs in facilitating the adherence, biofilm formation, stress resistance, and adjustment properties of probiotics in the host immune system.

**TABLE 5:** Exopolysaccharide (EPS) production by the probiotic isolates quantified by the phenol sulphuric acid assay.

Isolate	EPS concentration (µg/mL glucose eq.)
VITMS03	22.2
VITMS09	12.6
VITMS10	46.4
VITMS14	13.2
VITMS23	4.2
VITMS34	24.8
VITMS36	5.4
VITMS53	15.7
VITMS54	8
VITMS55	33.8
VITMS56	8.7
VITMS57	7.8
VITMS80	19.2

#### 3.4.4 Biofilm production

The ability of the probiotic isolates to produce biofilms was assessed by the CV assay (Supplementary Figure S4), where the amounts of biofilm formed were determined through absorbance measurements at 590 nm. From the measured values, the isolates were classified based on their biofilm–forming abilities; VITMS09 and VITMS10 were found to be the top biofilm formers, showing OD values greater than 1.0, as depicted in Figure 5D. These findings indicate strong cell surface adherence and high levels of extracellular matrix production. Such high degrees of biofilm formation imply that these strains are able to effectively persist and colonise the gut. VITMS55 was found to produce a moderate biofilm given its OD value of approximately 0.51, which indicates an intermediate level of bacterial adhesion to surfaces. The remaining isolates showed low OD values that correlated with weak or almost no biofilm formation. Therefore, probiotic strains of different origins evidently have highly variable capacities for biofilm formation, as observed experimentally in this study.

#### 3.4.5 Antibacterial activities of the postbiotic extracts

**FIGURE 6:**
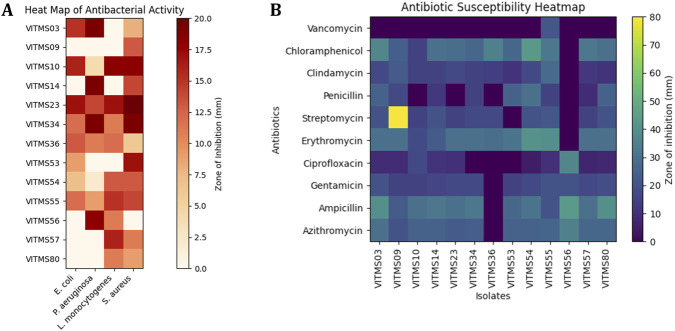
Heatmap analysis of the antibacterial activities and antibiotic susceptibilities of the probiotic isolates. The heatmaps depict the **(A)** antibacterial activities of the probiotic isolates against indicator pathogens *Pseudomonas aeruginosa* (MTCC 2582), *Escherichia coli* (MTCC 443), *Listeria monocytogenes* (MTCC 657), and *Staphylococcus aureus* (MTCC 3160) and **(B)** antibiotic susceptibility profiles against streptomycin (S), vancomycin (VA), clindamycin (CD), chloramphenicol (C), erythromycin (E), azithromycin (AZM), gentamicin (GEN), ciprofloxacin (CIP), ampicillin (AMP), and penicillin-G (P) expressed in terms of the diameter of the zone of inhibition (mm).

The antibacterial activities of probiotic preparations obtained from the probiotic isolates were measured by testing against indicator pathogens (Supplementary Table S2). The antibacterial effects were dependent on the bacterial strain, and only a small number of isolated bacteria exhibited notable inhibitory activities (Figure 6A). The isolates VITMS54, VITMS55, and VITMS56 showed good antibacterial activities, meaning that they contained bioactive antimicrobial compounds in their cell-free supernatants. The postbiotic-mediated pathogen inhibition of these isolates is also the highest among the tested strains. However, the other probiotic isolates did not show any measurable antibacterial activities under the test conditions, meaning that they had little or no production of antimicrobial postbiotic metabolites. The antibacterial activities detected herein demonstrate the functional diversity of postbiotic products among probiotic strains and hence the necessity for strain-specific screening to identify candidates for antimicrobial or gut-protective applications.

### 3.5 Identification of potential postbiotic-producing probiotics using 16S rRNA gene sequencing

**FIGURE 7:**
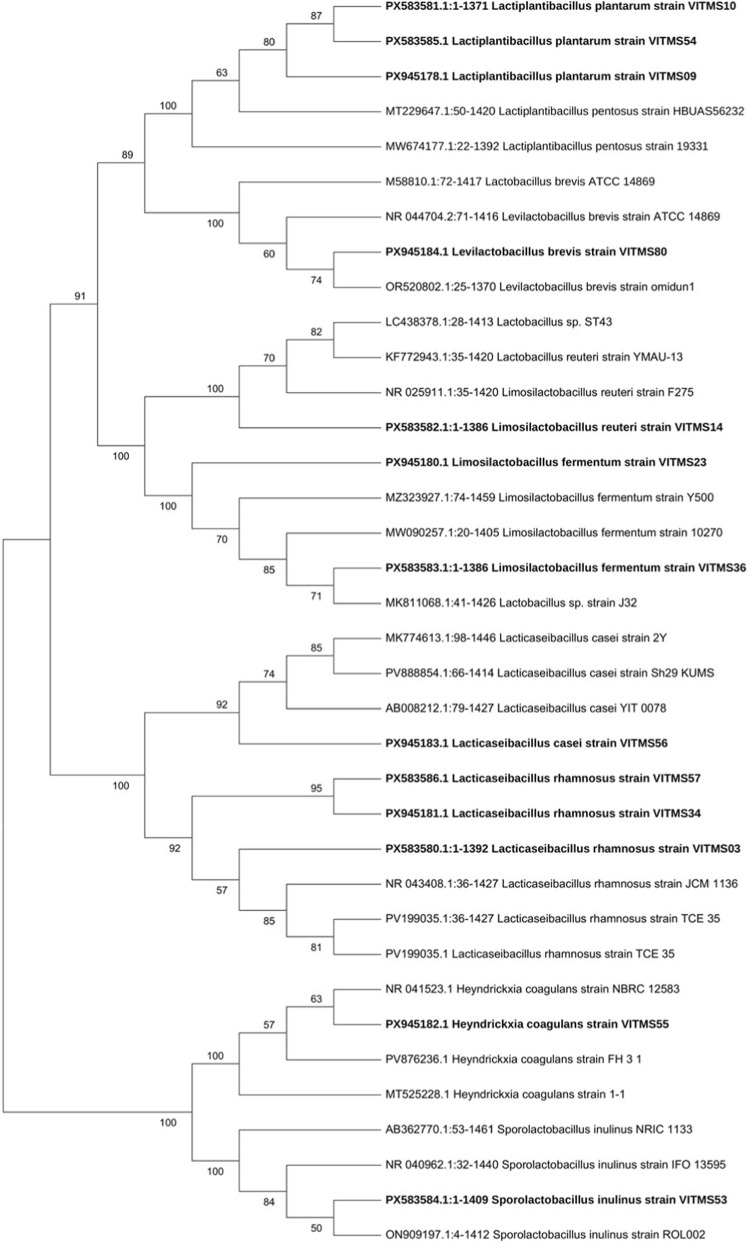
Phylogenetic relationships of the probiotic isolates based on 16S rRNA gene sequencing. The neighbour-joining phylogenetic tree based on the 16S rRNA gene sequences showed strong bootstrap support (500 replicates each) for most isolate clusters, with the bootstrap values ranging from 50% to 100%. All isolates identified in this study (phylogenetic tree shows bold strains without them) were grouped consistently with their closest reference strains and supported by high bootstrap values (≥85%) at the species level. The GenBank accession numbers for the isolates are indicated as prefixes to the names of the strains.

The postbiotic production potential of the selected probiotic isolates was identified by 16S rRNA gene sequencing. This analysis allowed taxonomic assignment of all isolates to the species level, thereby confirming their affiliations with well-known probiotic genera. The approved sequences were uploaded to the GenBank database under the accession numbers shown. The isolates identified are also presented in Table 6. VITMS03, VITMS34, and VITMS57 were identified as *Lacticaseibacillus rhamnosus* (accession numbers PX583580, PX945181, and PX583586, respectively); VITMS09, VITMS10, and VITMS54 were identified as *Lactiplantibacillus plantarum* (PX945178, PX583581, and PX583585, respectively); VITMS14 was identified as *Limosilactobacillus reuteri* (PX583582); VITMS23 and VITMS36 were identified as *Limosilactobacillus fermentum* (PX945180 and PX583583, respectively); VITMS80 was identified as *Levilactobacillus brevis* (PX945184); VITMS53 was identified as *Sporolactobacillus inulinus* (PX583584); VITMS55 was identified as *Heyndrickxia coagulans* (PX945182); VITMS56 was identified as *Lacticaseibacillus casei* (PX945183). These phylogenetic relationships of the isolates are presented in Figure 7.

**TABLE 6:** Partial molecular identification of the probiotic isolates having postbiotic production potential using 16S rRNA gene sequencing.

Bacterial isolate code	16S identified species	GenBank accession number
VITMS03	*Lacticaseibacillus rhamnosus*	PX583580
VITMS09	*Lactiplantibacillus plantarum*	PX945178
VITMS10	*Lactiplantibacillus plantarum*	PX583581
VITMS14	*Limosilactobacillus reuteri*	PX583582
VITMS23	*Limosilactobacillus fermentum*	PX945180
VITMS34	*Lacticaseibacillus rhamnosus*	PX945181
VITMS36	*Limosilactobacillus fermentum*	PX583583
VITMS53	*Sporolactobacillus inulinus*	PX583584
VITMS54	*Lactiplantibacillus plantarum*	PX583585
VITMS55	*Heyndrickxia coagulans*	PX945182
VITMS56	*Lacticaseibacillus casei*	PX945183
VITMS57	*Lacticaseibacillus rhamnosus*	PX583586
VITMS80	*Levilactobacillus brevis*	PX945184

The molecular identification results confirmed that most of the isolated strains were lactic acid bacteria and related spore-forming taxa known for probiotic properties and postbiotic production potential. These taxonomic identities are in line with functional data derived from the postbiotic stress tolerance and bioactivity tests, thus confirming the probiotic and postbiotic capacities of the selected isolates.

## 4 Discussion

The present study systematically explores culturing of probiotic bacteria isolated from the gut microbiomes of an underrepresented tribal population, with emphasis on the functional traits and inferred postbiotic-producing potential. The study focused on the gastric phase as a preliminary screening step; however, inclusion of the intestinal phases would provide a more comprehensive evaluation and will be considered in future studies. The findings of this work contribute to the growing body of evidence that non-industrialised human populations represent valuable reservoirs of microbiota with diverse metabolic capabilities, which may have become diminished in urbanised individual owing to dietary and lifestyle transitions. One of the key outcomes of this study is the successful isolation and screening of multiple Gram-positive bacterial strains exhibiting tolerance to gastrointestinal stressors, including acidic pH, bile salts, and osmotic conditions. These tolerance traits are widely regarded as essential prerequisites for probiotic functionality as they determine the ability of micro-organisms to survive the transit through the gastrointestinal tract. The observed variability among the isolates highlights the strain-specific nature of probiotic characteristics, which are consistent with previous reports emphasising that functional attributes cannot be generalised even within closely related taxa.

One of the central therapeutic goals in the treatment of dysbiosis-related disorders, such as inflammatory bowel disease, antibiotic-associated diarrhoea, and metabolic inflammation, is the restoration and maintenance of gut eubiosis (Guandalini, 2014). Probiotics help the body achieve this goal in many ways; they can cause the intestinal lumen to become more acidic, compete directly with pathogens to eliminate them, support epithelial barrier integrity, and also modulate the immune system of the host. *L. rhamnosus* GG is among the clinically validated examples of probiotics that have shown great performance in preventing antibiotic-associated diarrhoea as meta-analyses have documented the expected risk reduction as approximately 50%, thereby proving the importance of strain-specific probiotic characteristics (Xie et al., 2026). The present study, by its very design, has enabled the identification of several native probiotic isolates, including important ones like *L. rhamnosus* (VITMS03, VITMS34, and VITMS57), *H. coagulans* (VITMS55), and *L. plantarum* (VITMS09, VITMS10, and VITMS54), which demonstrate extraordinary abilities to withstand the harshness of gastric and biliary fluids while achieving survival percentages over 70%. This degree of durability is essential to actually survive the transit through the gastrointestinal tract and is often linked to bile salt hydrolase function, membrane alterations, and stress-response mechanisms. Interestingly, the levels of resilience demonstrated by our tribal gut isolates were on par with or even better than the tolerance levels of lactic acid bacteria from fermented foods and commercial probiotics disclosed in literature, thus accentuating that the functional abundances in indigenous guts may be moulded by their traditional way of life and diets (Darmastuti et al., 2021).

Effective probiotic functions are closely dependent on the abilities of the probiotic strains to attach to and stay within the intestinal environment. Increasing the cell surface hydrophobicity and auto-aggregation capacity of the probiotic strain can greatly help it achieve close contact with the intestinal epithelial cells. In turn, this facilitates biofilm formation by the probiotic bacteria, resulting in enhanced colonisation stability and pathogen exclusion. According to our isolate study, one method by which this occurs is that the leading isolates achieve hydrophobicity values in excess of 60% and demonstrate strong auto-aggregation. These two characteristics are just two of the many features that are directly correlated with decreased pathogen translocation and have also been shown to improve the barrier functions of intestinal epithelial in experimental models of inflammation (Akhtar et al., 2022; Hazarika et al., 2022; Carrera Marcolin et al., 2024). Consequently, these biologically relevant characteristics could be the basis of long-term host–microbe interactions that are essential for sustained probiotic efficacy. In addition to colonisation, another major avenue of probiotic–host communication is undoubtedly through metabolic output (Lin et al., 2023; Miao et al., 2024). The strains isolated herein were found to produce SCFAs of physiological relevance, namely acetates, propionates, and butyrates. These three SCFAs are the most recognised and important types for achieving intestinal homeostasis. By activating the G-protein-coupled receptors (GPR41 and GPR43), the SCFAs mediate anti-inflammatory effects, which also include enhancing Treg differentiation and inhibiting histone deacetylases. Together, these mechanisms enable immune tolerance and epithelial healing that have been clinically and experimentally evidenced (Ang and Ding, 2016; Ozma et al., 2022). For example, *L. plantarum* postbiotics have been demonstrated to lower disease activity and flares in patients with ulcerative colitis (Jung et al., 2022; Kim et al., 2022; Zhang et al., 2025).

It is of crucial note that the functional performances of these probiotic strains depend heavily on the diets that they are paired with. The conventional dietary choices of south Indian tribes, which are largely composed of millets, tubers, and resistant starch, offer selective substrates that favour lactobacilli and hence enable cross-feeding interactions with butyrate-producing taxa, such as *L. plantarum* (Anwesh et al., 2016; Hazarika et al., 2022). These trophic networks enhance the beneficial effects of primary fermenting microbes and help stabilise the ecosystem of the gut microbiota. There are similar reports regarding dietary and microbial synergies in other indigenous groups, which strengthen the idea that traditional diets enable gut microbiomes that are functionally resilient to stress (Nguyen et al., 2007; Ooi et al., 2021; Chang et al., 2022; Kim et al., 2022). Thus, the results of this study show that probiotic strains isolated from indigenous tribal populations have numerous desirable functional traits, including gastrointestinal resilience, good adhesion capacity, immunomodulatory metabolite production, and safety, which collectively render them as potential candidates for next-generation probiotic and postbiotic products. Finally, our study strongly supports the enormous therapeutic value of non-industrialised microbiomes that are yet to be realised and advocates their use in microbiome-based therapeutic strategies in the future.

## 5 Limitations of the study

The present study is primarily limited by its exclusively *in vitro* design and the absence of comprehensive metabolomic characterisation of the postbiotic compounds. The SCFA detection in this work was of a preliminary nature and not fully validated quantitatively. Additionally, the taxonomic identification based on 16S rRNA gene sequencing may not provide strain-level resolution. The potential influences of age-related variability on the composition of the gut microbiome were not specifically controlled and represent another limitation of this study. Future studies involving whole-genome sequencing, metabolomic profiling, and *in vivo* validations are therefore necessary to confirm the functional and therapeutic potential of the identified isolates. Notwithstanding these limitations, our study offers important preliminary insights into the functional potential of probiotic isolates derived from a traditionally underexplored population. The integration of culture-based isolation with multi-parameter functional screening provides a useful framework for identifying candidate strains with desirable properties. From a translational perspective, such strains may serve as a foundation for the development of postbiotic-based formulations or next-generation probiotics pending rigorous validation.

## 6 Future perspectives

Additional research efforts should be directed toward extracting and solving the structures of the most important cellular metabolites, such as SCFAs, EPSs, and proteolytic enzymes, along with exploring their effects in appropriate animal models of inflammation, metabolic disorders, and gut microbiota imbalance. At the same time, changes in large-scale fermentation processes are indispensable for promoting their utilisation in the preparation of functional foods, nutraceuticals, or disease treatments. Moreover, clinical evaluations and genome-resolved studies aimed at identifying new biosynthetic gene clusters will be necessary before such tribal-derived microbial resources can be used as precision postbiotic therapeutics. Based on these considerations, our study provides a firm basis for the efficient use of native microbiomes in next-generation management of gut health. We suggest that future studies should focus on whole-genome sequencing of the selected strains, detailed metabolomic profiling of the postbiotic compounds, and rigorous validation using *in vivo* models to establish the functional efficacy and safety of the isolates. Such approaches are essential to translate these findings into clinical or industrial applications in the future.

## 7 Conclusions

The present research work focuses on the isolation, characterisation, and functional validation of probiotics derived from stool samples of the Mulluvadi tribal population to identify a diverse community of native micro-organisms with strong postbiotic production capabilities. In this study, we successfully isolated 13 bacterial strains and thoroughly screened them for probiotic and postbiotic qualities through various assays, such as tolerance to the gastrointestinal environment, adhesion capabilities, enzymatic activities, antimicrobial properties, and immune-stimulatory effects. Among the bacterial isolates tested herein, strains of *L. plantarum* (VITMS10), *H. coagulans* (VITMS55), and *L. rhamnosus* (VITMS03) were the most impressive in terms of functionality (Table 7). These bacteria demonstrated excellent survival rates when exposed to environments simulating physiological gastric and biliary conditions (survival >70%), high cell surface hydrophobicity (>60%), strong auto-aggregation, and significant production of organic acids and SCFAs. Furthermore, they showed highly effective anti-inflammatory protease activities, as indicated by the large casein hydrolysis zones (15–20 mm), and significant membrane stabilisation effects through the HRBC assay (65%–82% protection), which are on par with the anti-inflammatory standards. The ability of the isolates to inhibit a wide range of pathogens by secreting antimicrobial substances is also a testimony to their biological importance.

**TABLE 7:** Comparative scoring of the postbiotic functional activities of selected probiotic isolates.

Probiotic isolates	Ability to produce SCFAs	Anti-inflammatory protease activity	Exopolysaccharide synthesis	Biofilm formation	Antibacterial activity toward indicator strains
VITMS10	+++	++++	++++	++++	++++
VITMS55	+++	+++	++++	+++	++++
VITMS03	+++	+	++	+++	++

Importantly, all of the leading isolates were found to be safe and not cause haemolysis, produce DNase, or show any undesirable resistance features. The deductions from our findings indicate a very high degree of functional diversity of the tribal gut microbiome and that the gut microbial communities of native populations may be good sources of probiotics and postbiotics with a greater range of functionalities than those isolated typically from urban or industrialised populations. The identification of probiotic bacteria with postbiotic-producing potential in this work highlights the utility of postbiotics as potential alternatives to traditional probiotic live cells since postbiotic preparations offer increased stability, less variability in host colonisation, and enhanced safety in immunocompromised and aged individuals.

## Data Availability

The datasets presented in this study can be found in online repositories. The names of the repositories and accession numbers can be found in the article/Supplementary Material.
