# Supplementary material for: Exploring the probiotic landscape in understanding postbiotics from indigenous bacteria isolated from the stool samples of a tribal population at Mulluvadi village, Tamil Nadu, India
Source: Front Pharmacol. 2026 May 18;17:1817531. doi: 10.3389/fphar.2026.1817531 (PMC13222833; doi:10.3389/fphar.2026.1817531)
Supplement: Supplementary file 1 [file Supplementaryfile1.docx]

Table 1. **Preliminary phenotypic screening of bacterial isolates from stool samples.**
Gram reaction, catalase activity, and oxidase test profiles of 112 isolates obtained from 25 stool samples.

| Isolates Obtained | Gram's Reaction | Catalase | Oxidase |
| --- | --- | --- | --- |
| VITMS01 | ₊ rods | ₋ | ₋ |
| VITMS02 | ₊ cocci | ₋ | ₊ |
| VITMS03 | ₊ rods | ₋ | ₋ |
| VITMS04 | ₊ rods | ₋ | ₋ |
| VITMS05 | ₊ rods | ₋ | ₋ |
| VITMS06 | ₊ rods | ₋ | ₋ |
| VITMS07 | ₊ rods | ₋ | ₋ |
| VITMS08 | yeast cells | ₊ | ₊ |
| VITMS09 | ₊ rods | ₋ | ₋ |
| VITMS10 | ₊ rods | ₋ | ₋ |
| VITMS11 | ₊ rods | ₋ | ₋ |
| VITMS12 | ₊ rods | ₋ | ₋ |
| VITMS13 | ₊ rods | ₋ | ₋ |
| VITMS14 | ₊ rods | ₋ | ₋ |
| VITMS15 | ₊ rods | ₋ | ₋ |
| VITMS16 | ₊ rods | ₋ | ₋ |
| VITMS17 | ₊ rods | ₋ | ₋ |
| VITMS18 | ₊ rods | ₋ | ₋ |
| VITMS19 | yeast cells | ₊ | ₊ |
| VITMS20 | yeast cells | ₊ | ₊ |
| VITMS21 | ₊ rods | ₋ | ₋ |
| VITMS22 | yeast cells | ₊ | ₊ |
| VITMS23 | ₊ rods | ₋ | ₋ |
| VITMS24 | yeast cells | ₊ | ₊ |
| VITMS25 | yeast cells | ₊ | ₊ |
| VITMS26 | yeast cells | ₊ | ₊ |
| VITMS27 | yeast cells | ₊ | ₊ |
| VITMS28 | yeast cells | ₊ | ₊ |
| VITMS29 | yeast cells | ₊ | ₊ |
| VITMS30 | yeast cells | ₊ | ₊ |
| VITMS31 | ₊ rods | ₋ | ₋ |
| VITMS32 | ₊ rods | ₋ | ₋ |
| VITMS33 | yeast cells | ₊ | ₊ |
| VITMS34 | ₊ rods | ₋ | ₋ |
| VITMS35 | ₊ rods | ₋ | ₋ |
| VITMS36 | ₊ rods | ₋ | ₋ |
| VITMS37 | yeast cells | ₊ | ₊ |
| VITMS38 | yeast cells | ₊ | ₊ |
| VITMS39 | yeast cells | ₊ | ₊ |
| VITMS40 | yeast cells | ₊ | ₊ |
| VITMS41 | yeast cells | ₊ | ₊ |
| VITMS42 | yeast cells | ₊ | ₊ |
| VITMS43 | ₊ rods | ₋ | ₋ |
| VITMS44 | ₊ rods | ₋ | ₋ |
| VITMS45 | ₊ rods | ₋ | ₋ |
| VITMS46 | ₊ rods | ₋ | ₋ |
| VITMS47 | yeast cells | ₊ | ₊ |
| VITMS48 | yeast cells | ₊ | ₊ |
| VITMS49 | ₊ rods | ₋ | ₋ |
| VITMS50 | ₊ rods | ₋ | ₋ |
| VITMS51 | ₊ rods | ₋ | ₋ |
| VITMS52 | ₊ rods | ₋ | ₋ |
| VITMS53 | ₊ rods | ₋ | ₋ |
| VITMS54 | ₊ rods | ₋ | ₋ |
| VITMS55 | ₊ rods | ₊ | ₋ |
| VITMS56 | ₊ rods | ₋ | ₋ |
| VITMS57 | ₊ rods | ₋ | ₋ |
| VITMS58 | ₊ cocci | ₋ | ₋ |
| VITMS59 | yeast cells | ₊ | ₊ |
| VITMS60 | yeast cells | ₊ | ₊ |
| VITMS61 | yeast cells | ₊ | ₊ |
| VITMS62 | ₊ rods | ₋ | ₋ |
| VITMS63 | ₊ rods | ₋ | ₋ |
| VITMS64 | ₋rods | ₋ | ₋ |
| VITMS65 | ₊ rods | ₋ | ₋ |
| VITMS66 | ₊ rods | ₋ | ₋ |
| VITMS67 | yeast cells | ₊ | ₊ |
| VITMS68 | ₊ rods | ₋ | ₋ |
| VITMS69 | yeast cells | ₊ | ₊ |
| VITMS70 | yeast cells | ₊ | ₊ |
| VITMS71 | yeast cells | ₊ | ₊ |
| VITMS72 | yeast cells | ₊ | ₊ |
| VITMS73 | yeast cells | ₊ | ₊ |
| VITMS74 | yeast cells | ₊ | ₊ |
| VITMS75 | yeast cells | ₊ | ₊ |
| VITMS76 | ₊ rods | ₋ |  |
| VITMS77 | yeast cells | ₊ | ₊ |
| VITMS78 | yeast cells | ₊ | ₊ |
| VITMS79 | ₊ rods | ₋ | ₋ |
| VITMS80 | ₊ rods | ₋ | ₋ |
| VITMS81 | yeast cells | ₊ | ₊ |
| VITMS82 | yeast cells | ₊ | ₊ |
| VITMS83 | yeast cells | ₊ | ₊ |
| VITMS84 | yeast cells | ₊ | ₊ |
| VITMS85 | ₊ rods | ₋ | ₋ |
| VITMS86 | yeast cells | ₊ | ₊ |
| VITMS87 | yeast cells | ₊ | ₊ |
| VITMS88 | ₊ rods | ₋ | ₋ |
| VITMS89 | ₊ rods | ₋ | ₋ |
| VITMS90 | ₊ rods | ₋ | ₋ |
| VITMS91 | yeast cells | ₊ | ₊ |
| VITMS92 | yeast cells | ₊ | ₊ |
| VITMS93 | yeast cells | ₊ | ₊ |
| VITMS94 | ₊ cocci | ₊ | ₊ |
| VITMS95 | ₊ cocci | ₋ | ₊ |
| VITMS96 | ₊ cocci | ₊ | ₊ |
| VITMS97 | ₊ cocci | ₊ | ₊ |
| VITMS98 | ₊ cocci | ₊ | ₊ |
| VITMS99 | ₊ rods | ₋ | ₊ |
| VITMS100 | ₊ cocci | ₊ | ₊ |
| VITMS101 | ₊ cocci | ₊ | ₊ |
| VITMS102 | ₊ cocci | ₊ | ₊ |
| VITMS103 | ₊ cocci | ₊ | ₊ |
| VITMS104 | ₊ rods | ₊ | ₊ |
| VITMS105 | ₊ cocci | ₊ | ₋ |
| VITMS106 | ₊ cocci | ₊ | ₊ |
| VITMS107 | yeast cells | ₊ | ₊ |
| VITMS108 | ₊ rods | ₊ | ₊ |
| VITMS109 | ₊ rods | ₊ | ₊ |
| VITMS110 | ₊ rods | ₊ | ₊ |
| VITMS111 | ₊ rods | ₋ | ₋ |
| VITMS112 | ₊ rods | ₋ | ₊ |


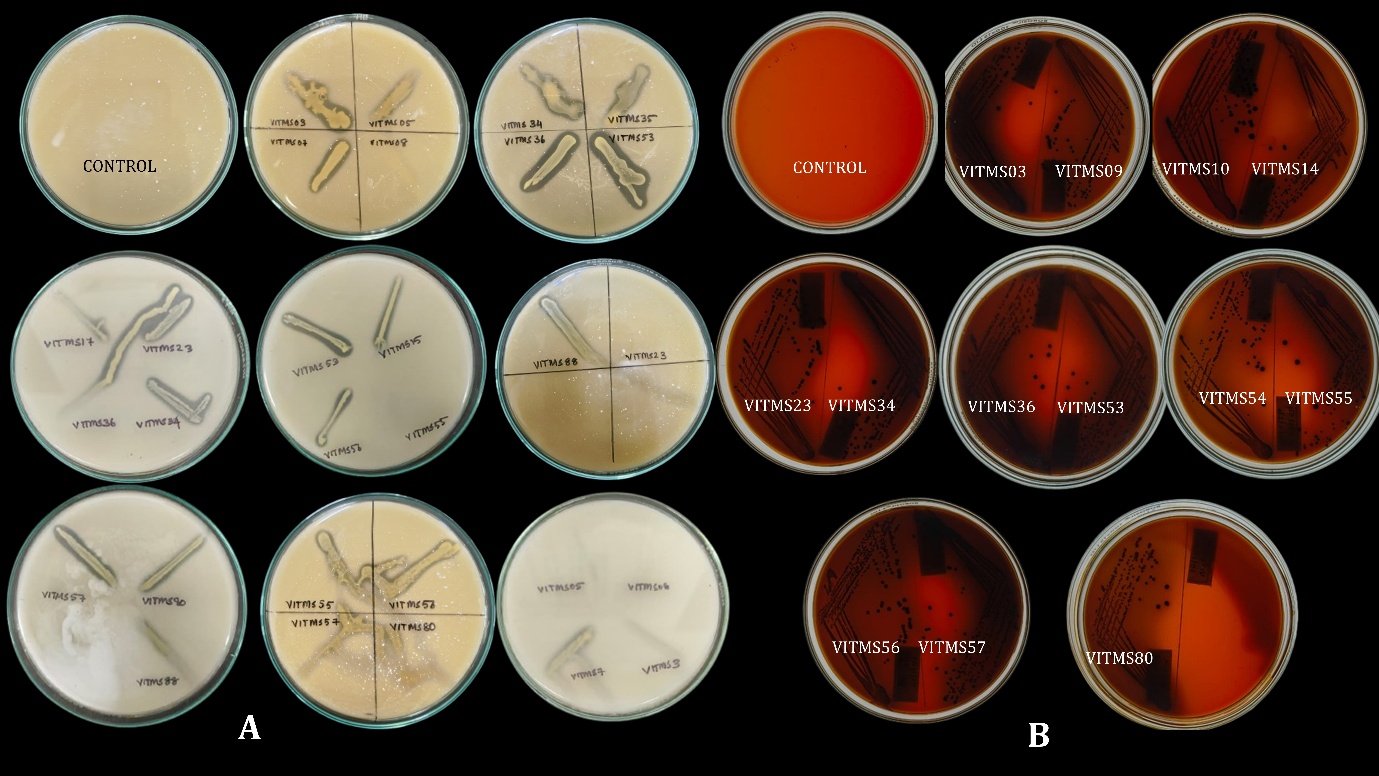


Figure 1. **Plate-based screening of CaCO₃ solubilization and EPS production.**
Representative images of CaCO₃ agar plates showing solubilization halos (**A**) and EPS-producing colonies on EPS-inducing medium (**B**) for isolates those only score positive.


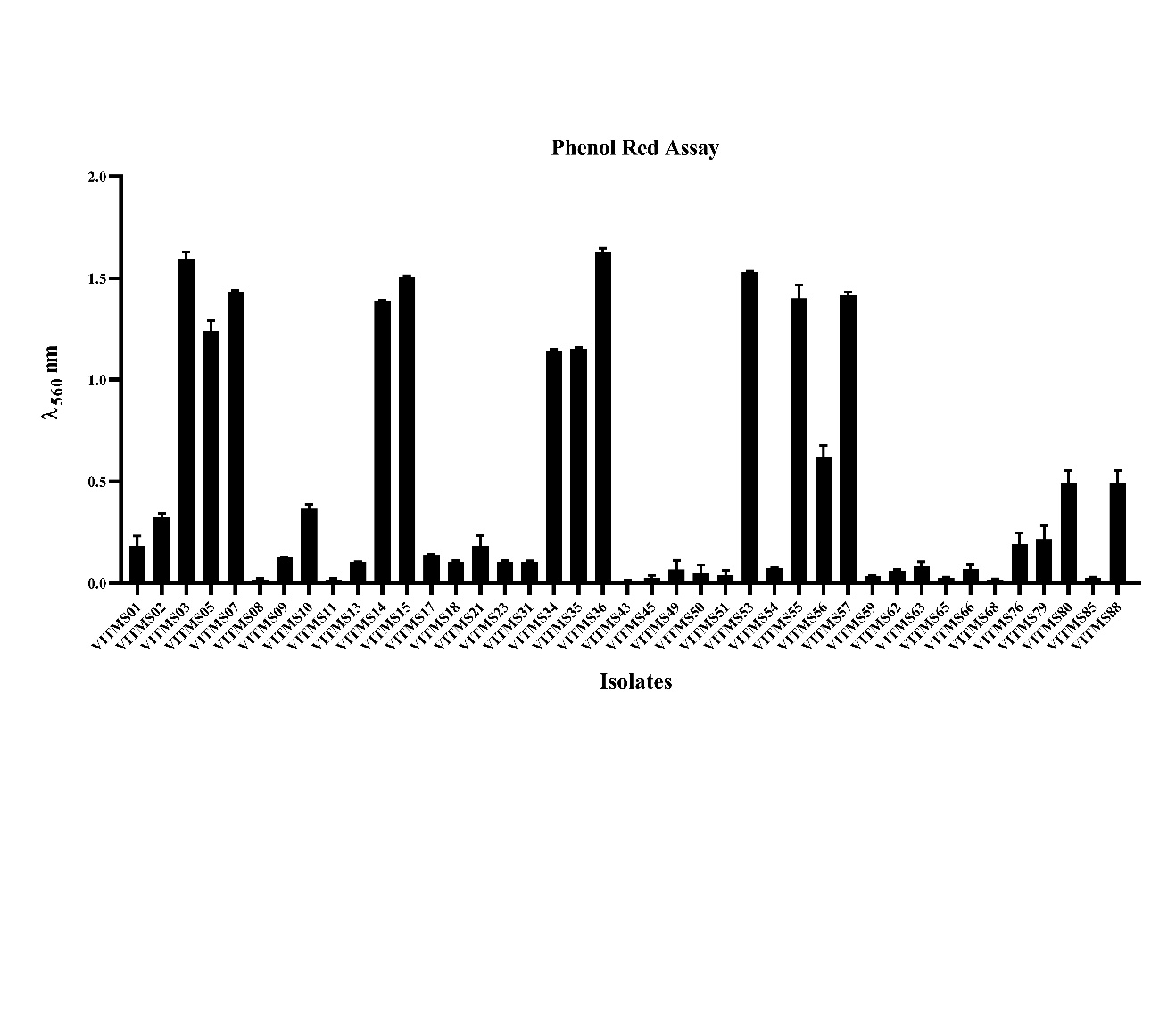


Figure 2. Results of phenol red assay for assessing the production of Organic Acids

Table 2. In vitro antibacterial activity of probiotic isolates. Inhibition zone diameters (mm) of probiotic isolates against selected Gram-positive and Gram-negative indicator pathogens.

| **Bacterial Isolates** | ***Escherichia coli* (MTCC 443)** | ***Pseudomonas aeruginosa* (MTCC 2582)** | ***Listeria monocytogenes* (MTCC 657)** | ***Staphylococcus aureus* (MTCC 3160)** |
| --- | --- | --- | --- | --- |
| VITMS03 | 15mm | 19mm | 0.0 | 8mm |
| VITMS09 | 0.0 | 0.0 | 0.0 | 13mm |
| VITMS10 | 16mm | 4mm | 18mm | 18mm |
| VITMS14 | 0.0 | 19mm | 0.0 | 14mm |
| VITMS23 | 17mm | 14mm | 17mm | 20mm |
| VITMS34 | 12mm | 19mm | 11mm | 19mm |
| VITMS36 | 13mm | 11mm | 12mm | 6mm |
| VITMS53 | 9mm | 0.0 | 0.0 | 17mm |
| VITMS54 | 7mm | 2mm | 13mm | 13mm |
| VITMS55 | 12mm | 9mm | 15mm | 14mm |
| VITMS56 | 0.0 | 18mm | 11mm | 0.0 |
| VITMS57 | 0.0 | 0.0 | 16mm | 11mm |
| VITMS80 | 0.0 | 0.0 | 11mm | 9mm |

Table 3. Zone of inhibition diameters (mm) and corresponding susceptibility classifications of probiotic isolates against vancomycin, chloramphenicol, clindamycin, penicillin-G, streptomycin, erythromycin, ciprofloxacin, gentamicin, ampicillin, and azithromycin, interpreted using established breakpoint criteria.


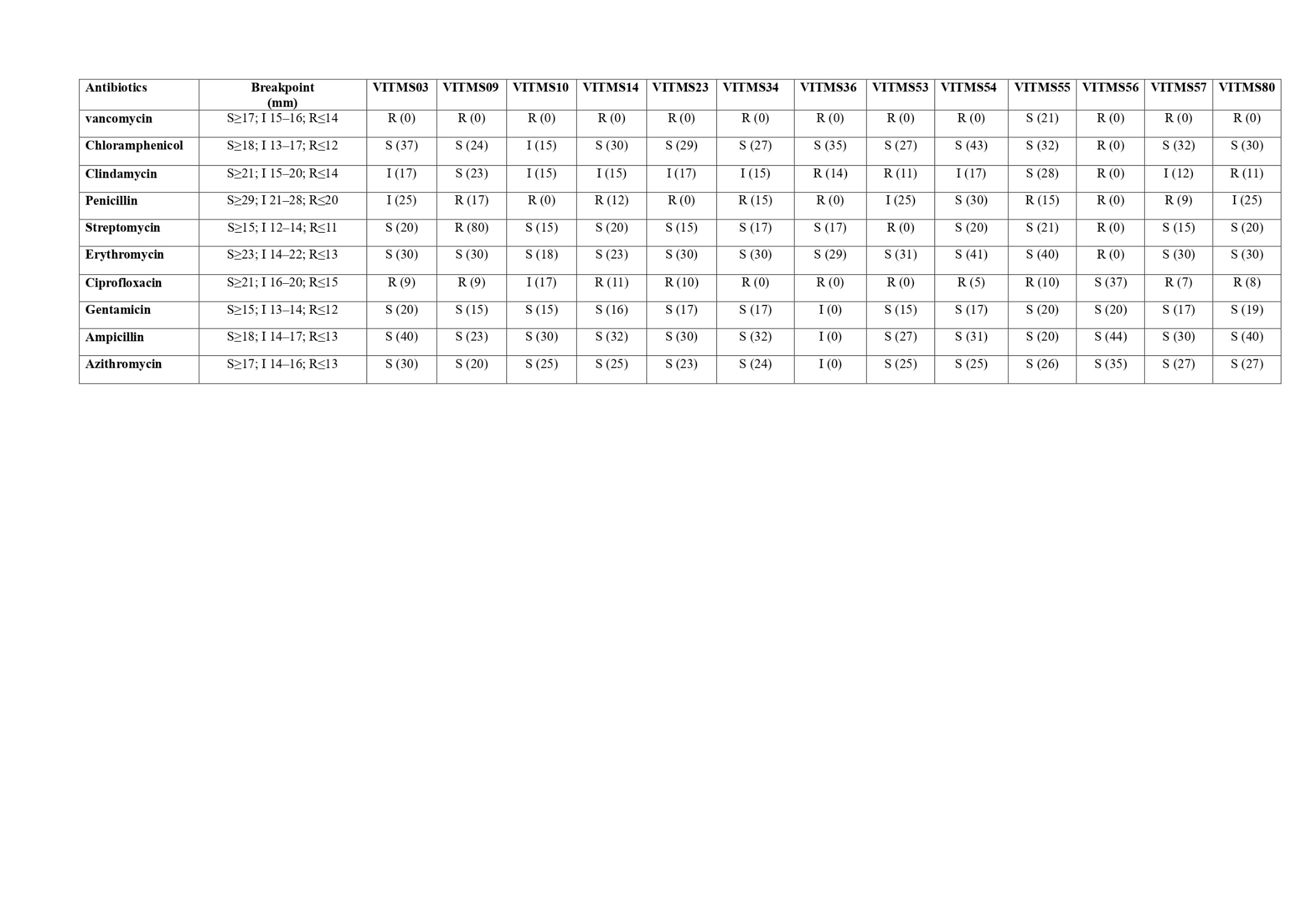

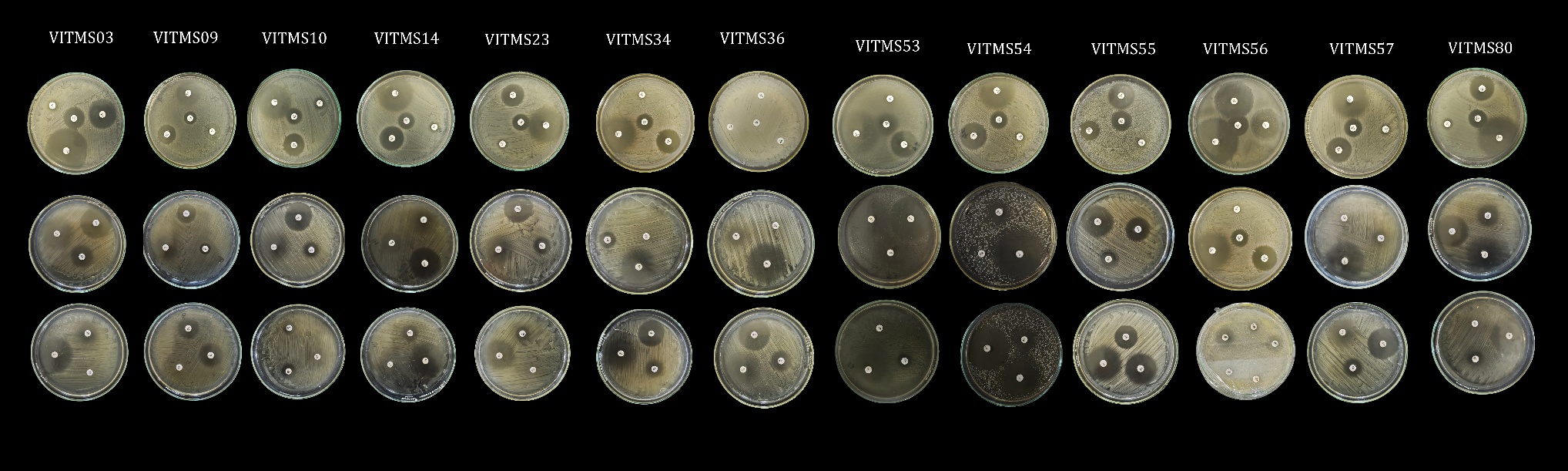


Figure 3. Disc diffusion–based antibiotic susceptibility patterns of probiotic isolates against ten antibiotics, expressed as zones of inhibition (mm) and categorized as susceptible (S), intermediate (I), or resistant (R) according to standard breakpoints.


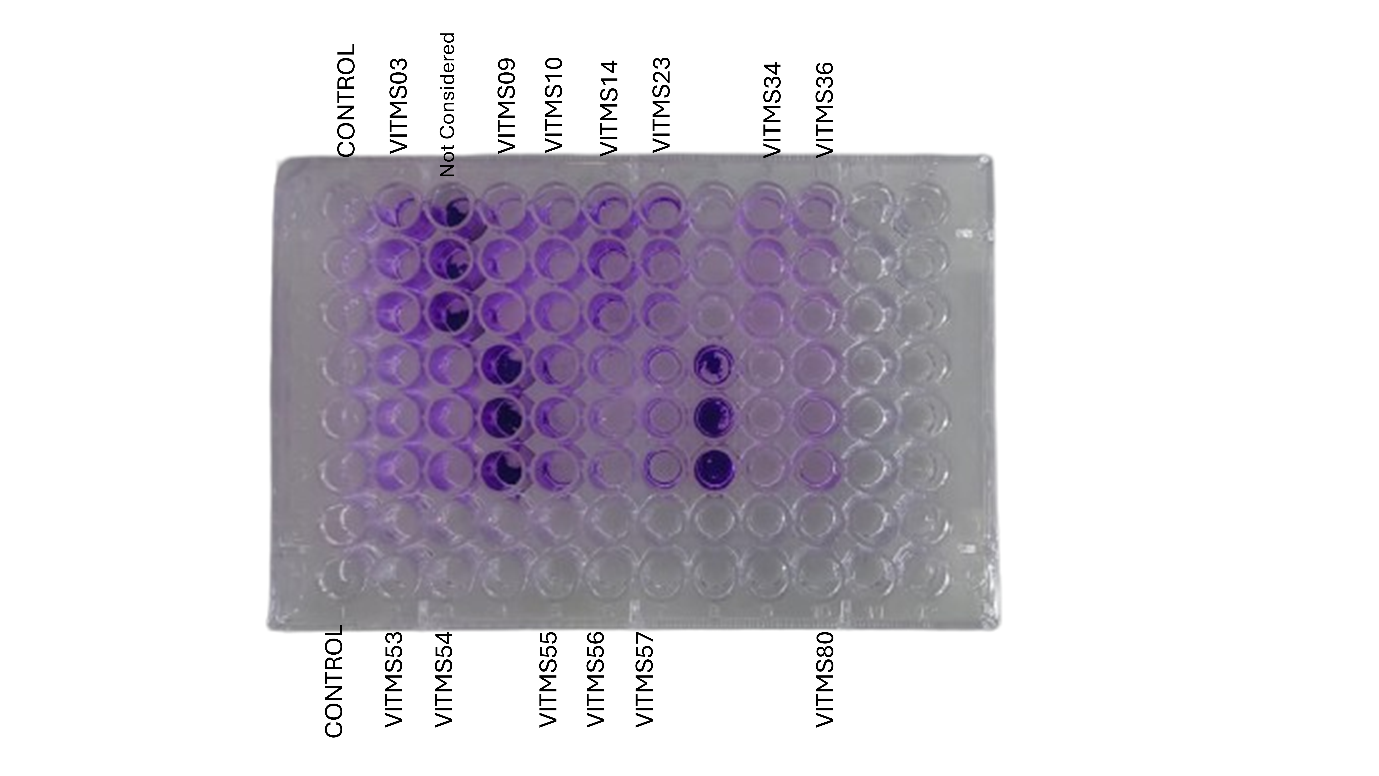


Figure 4. Biofilm formation quantified using a crystal violet microtiter plate assay and measured spectrophotometrically using a microplate (ELISA) reader. Data represent mean values obtained from triplicate experiments.
